# Female urethral diverticulum containing a urothelial carcinoma

**DOI:** 10.1590/0100-3984.2015.0115

**Published:** 2016

**Authors:** Rodolfo Mendes Queiroz, Paula Puty e Costa, Nara Yamada Fabril de Oliveira, Juliana Alves Paron, Eduardo Miguel Febronio

**Affiliations:** 1 Documenta - Hospital São Francisco, Ribeirão Preto, SP, Brazil.

Dear Editor,

We report the case of a 63-year-old black female who presented with complaints of difficulty in urinating and pollakiuria. She also reported an eight-month history of episodes of dysuria and hematuria. She had smoked for 10 years and had quit 30 years prior. She had three pregnancies, all with vaginal delivery, and had undergone total hysterectomy 17 years prior.

Magnetic resonance imaging (MRI) of the pelvis revealed, below the urinary bladder, a cystic formation involving the urethra, consistent with urethral diverticulum (UD), within which there was a solid component showing paramagnetic contrast enhancement, suggesting an expansive process. Communication with the urethra was well defined after a urethral catheter had been inserted. The diverticulum was surgically resected. On the basis of histological and immunohistochemical studies of the surgical sample, the patient was diagnosed with papillary urothelial diverticular carcinoma.

The reported prevalence of UD is 0.6-6.0%, and the condition is most common in women between 30 and 60 years of age^(1-8)^. Some studies have reported that the incidence of UD is higher in black individuals^(3,4,7)^. Typically, UD is underdiagnosed because, in most cases, the clinical profile is nonspecific^(2,4-6)^ and up to 20% of patients are asymptomatic^(4,8)^. The site most often affected is the middle third of the urethra, where the paraurethral glands (Skene's glands) are typically located, and 96% of diverticular orifices are posterolateral^(1-5,8)^. Most patients with UD have the acquired form, which probably arises from dilation/abscess in paraurethral glands. Other causes include trauma and surgery^(1-5,8)^. Typically measuring 0.2-1.6 cm^(5)^, UDs can be single or multiple, simple or multiloculated, and locally restricted or surrounding the urethra (in a "horseshoe" shape), with one or more (narrow or broad) orifices^(1,2)^. Differential diagnoses include cervical cysts, vaginal cysts, abscesses, tumors, urethral endometriosis, and ectopic ureterocele^(2,4,5)^.

**Figure 1:**
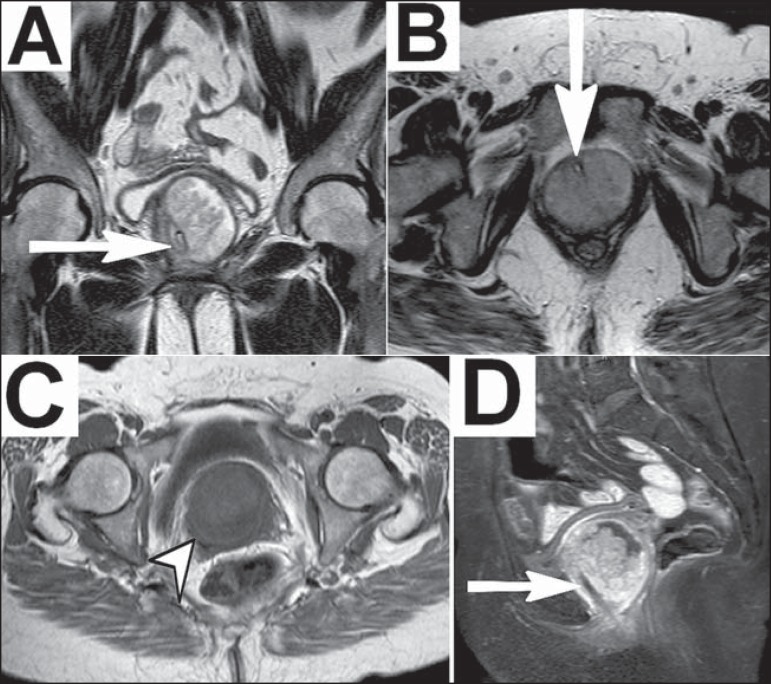
MRI of the pelvis. **A,B:** Coronal and axial T2-weighted sequences showing, respectively, cystic formation involving the urethra (showing hyperintense signal) and the UD, measuring approximately 5.5 × 5.3 × 5.4 cm. Within the diverticulum, an extensive solid expansive formation with intermediate signal can be seen. The urethral trajectory was identified after a urethral catheter had been inserted (arrows). **C:** A T1-weighted sequence showing the UD with hypointense signal (arrowhead). **D:** A T1-weighted sequence with fat saturation, after intravenous administration of paramagnetic contrast, highlighting the solid expansive component and the urethral catheter (arrow).

Clinical findings include the classic triad of dysuria (in 30-70% of cases), dyspareunia (in 10-25%) and postmicturition dribble (in 10-30%), as well as pollakiuria or urinary urgency (in 40-100%), urinary incontinence (in 32-60%), recurrent urinary tract infections (in 30-50%), hematuria (10-25%), and bulging in the anterior vaginal wall (in 35%), accompanied by purulent urethral discharge on palpation (in 12%)^(1-8)^. The chronic inflammation and urinary stasis seen in UDs result in complications^(3,4)^, including calculi (in 1.5-10%) and malignant tumors^(1-8)^. As for the tumors, UDs are responsible for less than 5% of all urethral neoplasms^(4,8)^, fewer than 200 cases having been reported^(1,2,7,8)^. Malignancies in UD include adenocarcinoma, in 49-61% of cases, transitional cell carcinoma, in 27-30%, and squamous cell carcinoma, in 10-12%^(1-5)^.

Diagnostic imaging methods include the following: voiding cystourethrography-a technically simple method, with an accuracy of 85%, that demonstrates the diverticulum through contrast and identification of filling gaps suggestive of calculi or tumors, although it uses an iodized agent might not detect UDs with small orifices^(1,4,5)^; double-balloon urethrography-a method with an accuracy of 90%, showing findings similar to those cystourethrography, with the disadvantage of being invasive and complex^(1,4,5)^; ultrasound-a method with excellent accuracy (near 100%) when intraurethral (highly invasive) or translabial/transperineal (less invasive) that characterizes the cystic formations and any vascularized solid content, although examinerdependent and limited in the evaluation of collapsed UDs^(1,4,5)^; computed tomography-a method that is useful in identifying calculi and tumors (evident solid components), albeit with low sensitivity for small UDs^(1,4,5)^; and MRI-the method of choice, with near 100% sensitivity, which is noninvasive, with excellent contrast between tissues and discrimination of the complexity of the structures, capable of detecting small UDs and identifying neoplasms^(1,2,4-6,8)^. In T-2 weighted MRI sequences, UDs show hyperintense signals, although they can be hypointense if they have thick content^(1,2,4,6)^. Solid tumor components present as vegetative lesions with intermediate signals on T1- and T2-weighted sequences, potentially restricting the diffusion, and show significant enhancement after intravenous administration of contrast^(1,2)^.
